# Significant Diagnostic and Prognostic Value of FLAD1 and Related MicroRNAs in Breast Cancer after a Pan-Cancer Analysis

**DOI:** 10.1155/2021/6962526

**Published:** 2021-07-21

**Authors:** Mei Mei, Wenting Song, Yingjun Wang, Mingzhi Zhang

**Affiliations:** ^1^Department of Oncology, The First Affiliated Hospital of Zhengzhou University, Zhengzhou, China; ^2^The Academy of Medical Sciences, Zhengzhou University, Zhengzhou, China

## Abstract

The identification of biomarkers plays an important role in the diagnosis and prognosis of cancers. In this study, we explored the diagnostic and prognostic value of the *FLAD1* expression across pan-cancer analysis from online databases (Oncomine, cBioPortal, Breast Cancer Gene-Expression Miner, UALCAN, GEO, BCIP, TNMplot, ENCORI, Kaplan-Meier Plotter, and LinkedOmics). We found that *FLAD1* was overexpressed in a number of different kinds of cancers, especially in breast cancer, and higher *FLAD1* expression level was associated with the HER+, p53 mutant, node-involved, NPI stage 3, basal-like, and triple-negative groups compared with the other subgroups of breast cancer. The *FLAD1* expression levels were higher in patients that were 21–40 years old than those in patients of other ages and were higher in the African-American group than in the Caucasian group. We also analyzed the *FLAD1-*related microRNAs and their prognostic values in breast cancer. This study highlights the significance of *FLAD1* in cancers and provides evidence for its potential as a biomarker for the diagnosis and prognosis of cancers.

## 1. Introduction

Breast cancer is the most common malignancy in women and the most common cause of cancer-related deaths in less-developed countries [1]. Approximately 2.1 million newly diagnosed female breast cancer cases were reported worldwide in 2018 [2]. Breast cancer has various subtypes that differ in histopathology, biology, and response to systemic treatment [3]. Despite the rapid development of new technologies and treatments, the identification of biomarkers for diagnosis and prognosis is still in the early research phase [4].

Flavin adenine dinucleotide synthetase 1 (*FLAD1*), also known as FAD1, is located on chromosome 1 [5] at 1q21.3 [6] (https://www.genecards.org/). It encodes flavin adenine dinucleotide synthase (FADS), which contains an N-terminal molybdopterin-binding (MPTb) domain and a C-terminal domain sufficient to catalyze FAD synthesis [7]. The *FLAD1* expression was previously reported to be upregulated in hepatocellular carcinoma and is considered to be related to hepatitis B virus infection [8]. Another study reported that *FLAD1*, as well as three other genomic markers, *DBN1*, *CACNB3*, and *CCND2*, could serve as a novel prognostic model of stage I-III non-small-cell lung cancer [9]. The *FLAD1* expression has also been shown to be upregulated in gastric cancer [10] and breast cancer [11].

With the development of high-throughput technology, the relationships between oncogene expressions and clinical factors have become obvious. However, the diagnostic and prognostic significance of *FLAD1* is unclear. Therefore, in this study, we searched for relevant data from online databases to determine the diagnostic and prognostic value of the abnormal expression of *FLAD1* and related miRNAs in pan-cancer analysis, especially in breast cancer. These results have implications for the development of new molecular biomarkers in breast cancer and provide evidence for the clinical value of *FLAD1*.

## 2. Materials and Methods

### 2.1. Oncomine Database

Oncomine is an online database [12] (http://www.oncomine.org) with sequencing and bioinformatic data for 715 datasets and 86,733 samples. We analyzed *FLAD1* in several kinds of tumors and selected 656 datasets. The thresholds were set as the following: *p* value (0.0001), fold change (2), gene rank (top 10%), and data type (all).

### 2.2. cBioPortal with The Cancer Genome Atlas (TCGA) Dataset

The cBioPortal for Cancer Genomics [13, 14] (http://www.cbioportal.org) includes large scale of cancer genomic dataset that can be visualized and analyzed online. We performed the analyses on the dataset “TCGA, PanCancer Atlas,” and the threshold was set as value ±2.

### 2.3. Breast Cancer Gene-Expression Miner (bc-GenExMiner) v4.6

bc-GenExMiner v4.6 (http://bcgenex.ico.unicancer.fr/BC-GEM/GEM-Accueil.php) [15–17] is a statistical mining tool of published annotated transcriptomic data. Statistical analyses are provided, including analyses of targeted expression, exhaustive expression, customized expression, targeted prognosis, exhaustive prognosis, molecular subtype prognosis, basal-like/TNBC prognosis, targeted gene correlations, exhaustive gene correlations, gene ontology, and gene correlations by chromosomal location. These data can be classified according to clinical and pathologic parameters.

### 2.4. ENCORI

The Encyclopedia of RNA Interactomes (ENCORI), (http://starbase.sysu.edu.cn/index.php), previously known as starBase v2.0 [18], is a public platform often used to analyze the interaction between mRNAs and noncoding RNAs among 23 species.

### 2.5. Cytoscope 3.8.2

The miRNA–mRNA network was drawn using Cytoscape 3.8.2 (http://www.cytoscape.org/) [19], which constructs complicated networks from original data.

### 2.6. TNMplot

TNMplot [20] (https://www.tnmplot.com/) is an online tool for the differential gene expression analysis among tumors.

### 2.7. UALCAN Analysis

UALCAN [21] (http://ualcan.path.uab.edu) uses related resources to analyze transcriptome data for 31 cancer types. It provides valuable data about genes or targets associated with clinical parameters.

### 2.8. GEO Datasets

GEO datasets (https://www.ncbi.nlm.nih.gov/gds/) contains original gene expression datasets, including raw data of sequencing, microarray, and platform information.

### 2.9. BCIP

BCIP (Breast Cancer Integrative Platform) [22] (http://www.omicsnet.org/bcancer/database) is a platform with gene expression, histopathological features, and clinical information of breast cancer samples.

### 2.10. Kaplan–Meier Plotter

Kaplan–Meier Plotter [23] (http://www.kmplot.com) is an online visualization tool for survival data for breast, lung, ovarian, liver, and gastric cancer. A *p* value of <0.05 was considered statistically significant.

### 2.11. LinkedOmics Analysis

The LinkedOmics database [24] (http://www.linkedomics.org) is a publicly available platform with data for 32 TCGA cancer types. Differentially expressed genes correlated with *FLAD1* in the TCGA breast invasive carcinoma cohort were identified. Results were analyzed using Spearman's correlation coefficients.

## 3. Results

### 3.1. *FLAD1* Overexpression in Pan-Cancer Analysis

**Figure 1:**
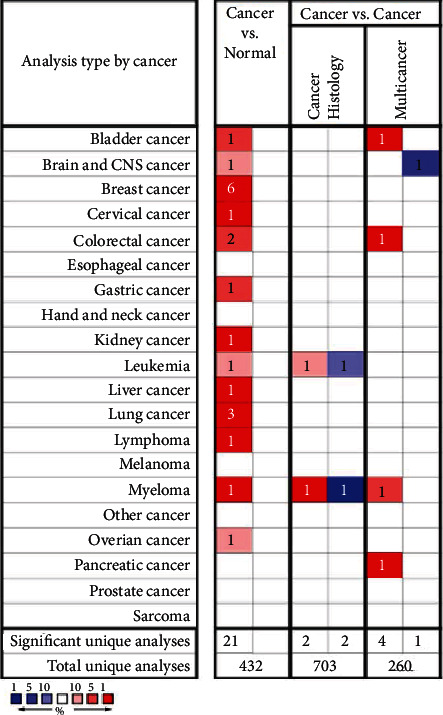
The transcription level of the FLAD1 in 20 types of human cancers in Oncomine.

**Figure 2:**
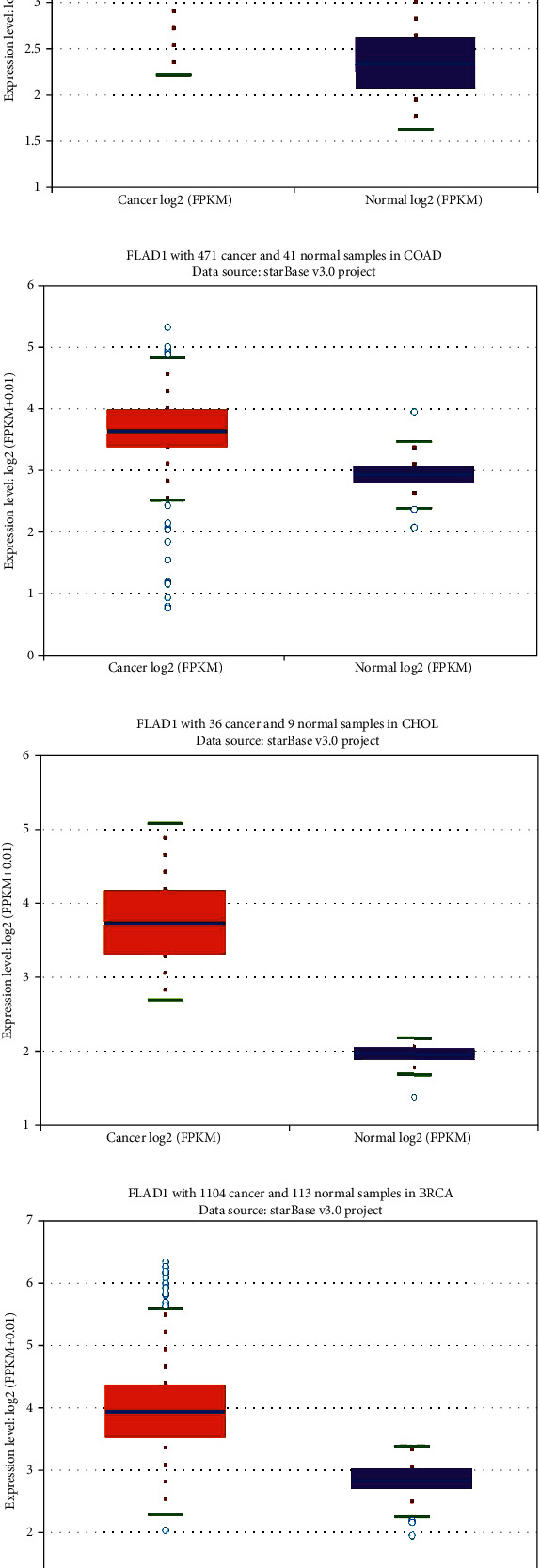
The transcription level of *FLAD1* in various cancer types in ENCORI: (a) kidney renal papillary cell carcinoma (KIRP), (b) head-neck squamous cell carcinoma (HNSC), (c) esophageal cancers (ESCA), (d) colon adenocarcinoma (COAD), (e) cholangiocarcinoma (CHOL), (f) breast invasive carcinoma (BRCA), (g) bladder urothelial carcinoma (BLCA), and (h) liver hepatocellular carcinoma (LIHC). *p* < 0.001 in all figures.

**Figure 3:**
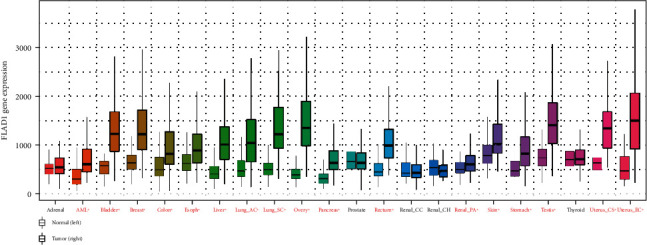
Boxplots of the *FLAD1* gene expression in 22 tumor types. Significant differences by a Mann–Whitney *U* test are marked with asterisk and red color (^∗^*p* < 0.01). Note: AML: acute myeloid leukemia.

We first analyzed the *FLAD1* expression in human cancers and found that it was overexpressed in six breast cancer datasets in the Oncomine database (Figure 1). We also found some evidence for the abnormal expression of *FLAD1* in various cancers in ENCORI [18]. A significant difference in the *FLAD1* expression between cancer samples and normal tissue samples was identified for eight cancer types (Figure 2, *p* < 0.001), including kidney renal papillary cell carcinoma, head-neck squamous cell carcinoma, esophageal cancers, colon adenocarcinoma, cholangiocarcinoma, breast invasive carcinoma, bladder urothelial carcinoma, and liver hepatocellular carcinoma. TNMplot [20] was further used for pan-cancer analysis of *FLAD1,* including 56,938 samples showing that *FLAD1* is highly expressed in acute myeloid leukemi, bladder cancer, breast cancer, colon cancer, esophageal cancer, liver cancer, lung adenocarcinoma, lung squamous cell cancer, ovarian cancer, pancreatic cancer, rectum cancer, kidney renal papillary cell carcinoma, skin cancer, stomach cancer, testicular cancer, uterine carcinosarcoma, and uterine corpus endometrial carcinoma (Figure 3, *p* < 0.001).

### 3.2. Frequency and Type of FLAD1 Alterations in Breast Cancer

We used cBioPortal to determine the type and frequency of *FLAD1* alterations in 106 of 996 patients with breast cancer. Only 10 cases were with mutation while 5 cases had missense mutations, 4 cases had truncation mutations, and 1 had SV/Fusion. Thus, amplification may be the most common type of *FLAD1* alteration in breast cancer.

### 3.3. Diagnostic Value and Related Clinical Features of the *FLAD1* Expression in Breast Cancer

**Figure 4:**
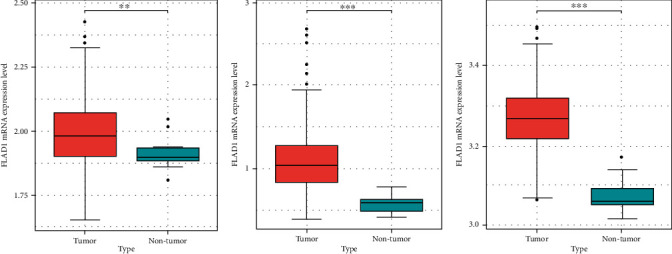
The *FLAD1* expression in breast cancers samples and normal controls from GEO. (a) GSE10797, *p* = 0.0067. (b) GSE22820, *p* < 0.001. (c) GSE54002, *p* < 0.001. ^∗^*p* < 0.05; ^∗∗^*p* < 0.01; ^∗∗∗^*p* < 0.001.

**Figure 5:**
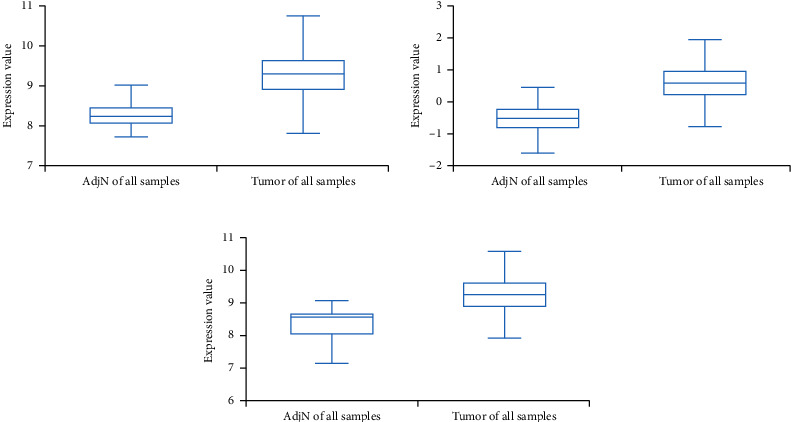
The *FLAD1* expression in breast cancers samples and normal controls from BCIP. (a) Dataset “Metabric”, *p* < 0.001. (b) Dataset “TCGA_Agilent”, *p* < 0.001. (c) Dataset “GSE5364_GPL96”, *p* = 0.008.

We then analyzed the *FLAD1* expression in three GEO datasets (GSE10797 [25], GSE22820 [26], GSE54002 [27]) and in BCIP (“Metabric,” [28] “TCGA_Agilent,” “GSE5364_GPL96” [29]). These results confirmed that the expressions levels of *FLAD1* in breast cancer groups are higher than that in normal tissue groups (Figures 4 and 5).

**Figure 6:**
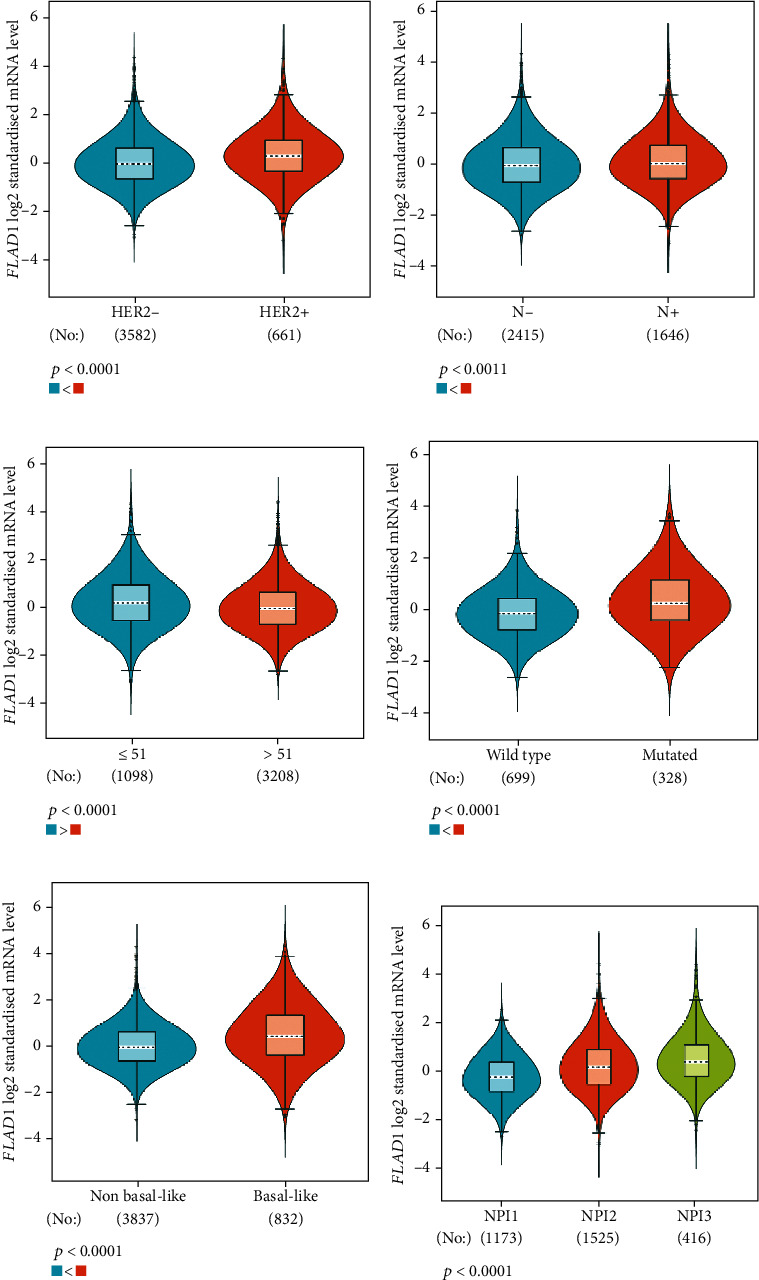
Violin plots showed the FLAD1 mRNA expression in subgroups of patients with breast cancer (bc-GenExMiner). (a) Expressions between HER2 (-) and HER2 (+). (b) Expression related to nodal status. (c) Expressions between age ≤ 51 and age > 51. (d) Expressions between p53 wild type and mutation. (e) Expressions between basal-like and nonbasal-like status. (f) Expression among NPI 1, 2, and 3.

The analysis using bc-GenExMiner showed that the ER-positive and PR-positive groups had lower *FLAD1* expression (*p* < 0.001) in line with a previous study [11]. However, the HER2-positive groups showed higher *FLAD1* expression (Figure 6(a), *p* < 0.001). Nodal status (Figure 6(b)*p* = 0.0011) and age (Figure 6(c), *p* < 0.001) were also related to the *FLAD1* expression. In addition, the p53 mutated group showed higher *FLAD1* expression (Figure 6(d), *p* < 0.001). The basal-like group showed higher *FLAD1* expression levels than those found for samples with a nonbasal-like status (Figure 6(e), *p* < 0.001). Similarly, the triple-negative group showed higher mRNA expression levels than those in the nontriple-negative group. Moreover, the expression level of *FLAD1* sequentially increased in advanced stages based on the NPI (Figure 6(f), *p* < 0.001).

**Figure 7:**
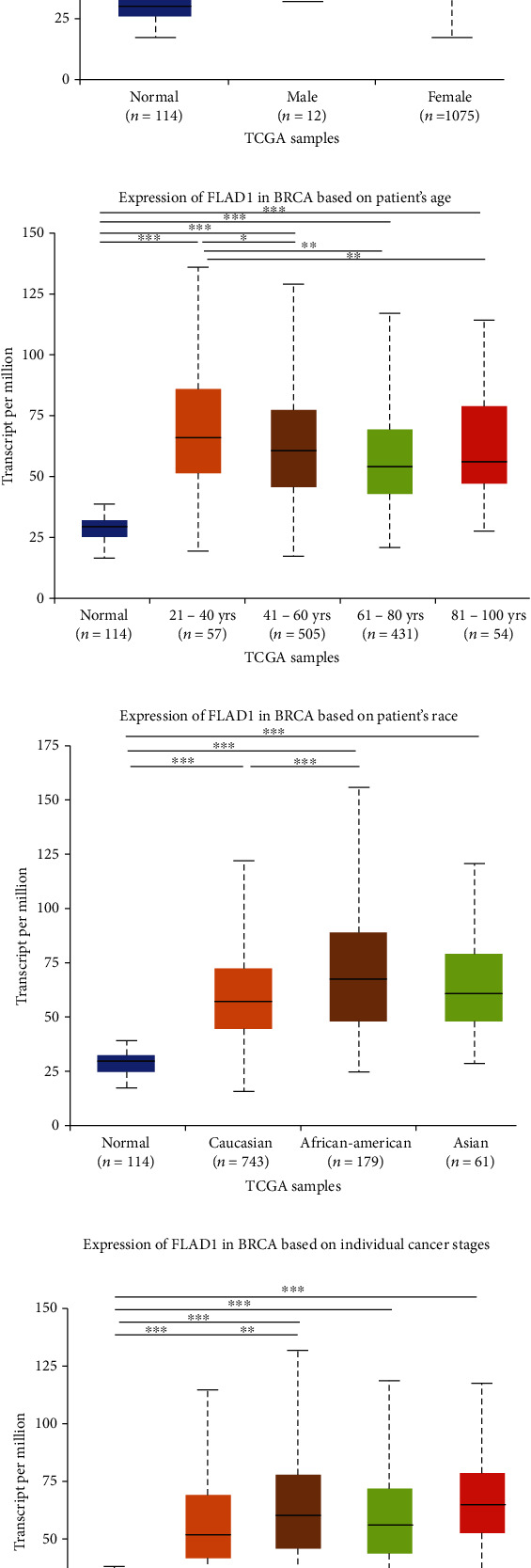
Boxplots showed FLAD1 transcription in subgroups of patients with breast invasive carcinoma (UALCAN). (a) Comparison between the normal group and cancer group. (b) Comparison between the normal group and different genders in patients' group (male and female). (c) Comparison between the normal group and different ages in patients' group (21–40, 41–60, 61–80, or 81–100 years). (d) Comparison between the normal group and different races in patients' group (Caucasian, African-American or Asian). (e) Comparison between the normal group and different stages in patients' group (stages 1, 2, 3, or 4). (f) Comparison between the normal individuals and different subclasses in patients' group (luminal, HER2-positive, and triple-negative). Data are mean ± SE. ^∗^*p* < 0.05; ^∗∗^*p* < 0.01; ^∗∗∗^*p* < 0.001.

UALCAN was also used to reveal the clinical parameters related to *FLAD1* in breast invasive carcinoma. The overall *FLAD1* mRNA expression level was significantly (*p* < 0.001) higher in the breast cancer group than in the healthy donors (Figure 7(a); median transcripts per million (TPM) of 58.593 and 29.68, respectively). There was no significant difference in the *FLAD1* levels according to gender (Figure 7(b), median TPM 58.821 for men and 58.519 for women, respectively; *p* = 0.818); although, significant differences were found for age (Figure 7(c)); the median TPM for patients who were 21–40 years old (median TPM = 66.653) was higher than those of other age groups: 41–60 years (median TPM = 60.918, *p* = 0.016), 61–80 years (median TPM = 54.837, *p* = 0.002), and 81–100 years (median TPM = 55.355, *p* = 0.003). Significant differences were also found with respect to race, with significantly higher expression levels being observed in African-American patients (median TPM = 68.061) than in Caucasian (median TPM = 56.558) and Asian (median TPM = 60.17) patients (*p* < 0.001; Figure 7(d)). There were no differences observed in the comparisons of the patients from the Caucasian vs. Asian (*p* = 0.078) or African-American vs. Asian (*p* = 0.332) groups. The *FLAD1* expression level was higher for all stages compared with the normal group. However, a significant difference (*p* = 0.003) between the stages was only found for stage 1 (median TPM = 52.445) and stage 2 (median TPM = 60.417), with no significant differences for the other comparisons: stage 1 vs. stage 3 (median TPM = 56.524), *p* = 0.135; stage 1 vs. stage 4 (median TPM = 66.849), *p* = 0.064; stage 2 vs. stage 3, *p* = 0.255, stage 2 vs. stage 4, *p* = 0.492; and stage 3 vs. stage 4, *p* = 0.320 (Figure 7(e)). In addition, the *FLAD1* expression levels in the luminal subclass (median TPM = 56.156) were also significantly lower than those in the HER2-positive (median TPM = 67.728) and triple-negative (median TPM = 76.715) groups (Figure 7(f), *p* = 0.003 and *p* < 0.001, respectively), whereas no significant difference was found between the *FLAD1* expression levels in the HER2-positive and triple-negative groups (*p* = 0.232).

### 3.4. Prognostic Value of the *FLAD1* Expression in Breast Cancer and Other Cancers

**Figure 8:**
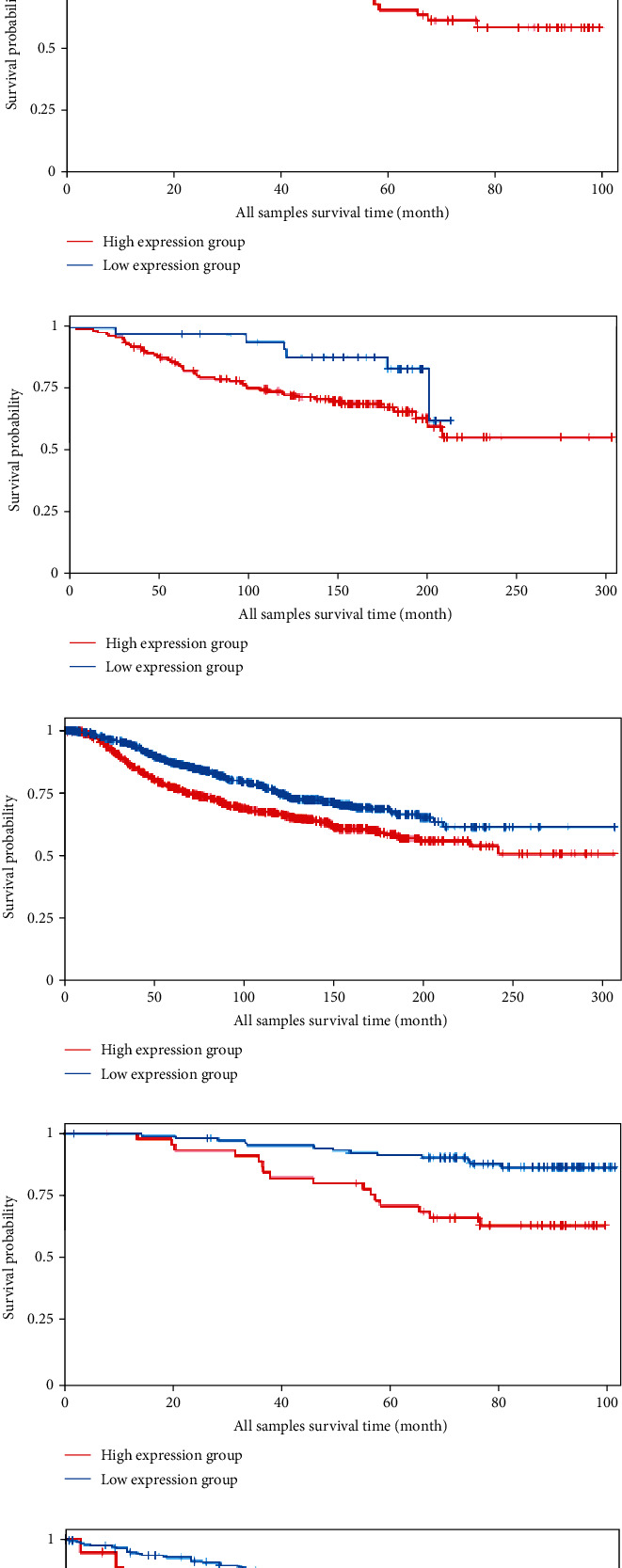
Overall survival (OS) and disease-specific survival (DS) between the high and low expression of the *FLAD1* group. (a) OS from dataset “Metabric” [28], *p* = 0.003. (b) OS from dataset “GSE1456_GPL96” [30], *p* = 0.004. (c) OS from dataset “GSE7390_GPL96” [31], *p* = 0.046. (d) DS from dataset “Metabric”, *p* < 0.001. (e) DS from dataset “GSE1456_GPL96”, *p* < 0.001. (f) DS from dataset “GSE3494_GPL96” [32], *p* = 0.004.

We analyzed the survival data from the BCIP, which showed that the low expression of *FLAD1* is associated with longer overall survival (OS) and disease-specific survival (Figure 8).

**Figure 9:**
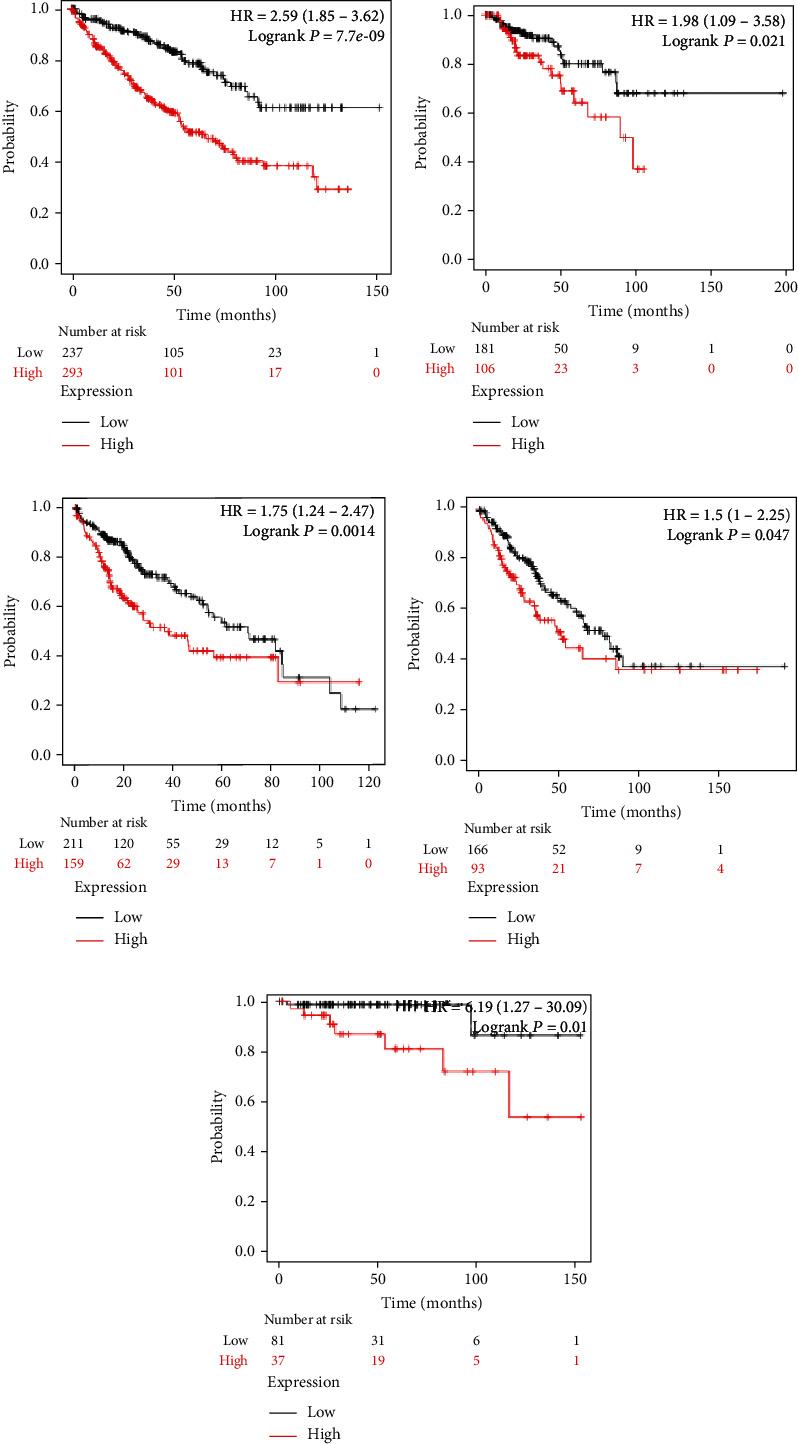
Survival analyses of the FLAD1 expression in cancers: (a) kidney renal clear cell carcinoma, (b) kidney renal papillary cell carcinoma, (c) liver hepatocellular carcinoma, (d) sarcoma, and (e) thymoma.

We also analyzed the relationships between the *FLAD1* expression and OS in a number of different kinds of cancers and found that the *FLAD1* overexpression was related to a poorer OS in five types of cancers: kidney renal clear cell carcinoma, kidney renal papillary cell carcinoma, liver hepatocellular carcinoma, sarcoma, and thymoma (Figure 9).

### 3.5. *FLAD1*-Related miRNA Network and Prognostic Value

**Figure 10:**
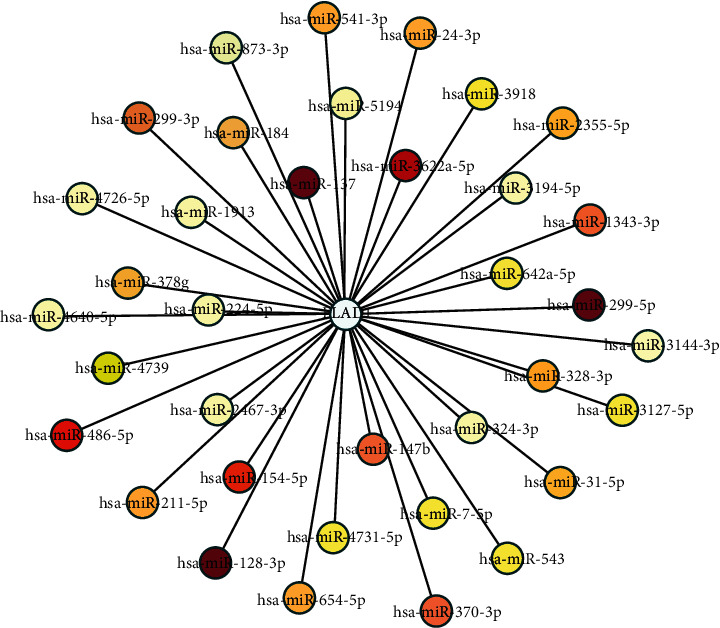
FLAD1-related miRNAs. Darker color (red) represents more experimental evidence.

We used ENCORI to analyze the mRNA–miRNA interactions and then used cystoscope to visualize the network. The related miRNAs were identified included hsa-miR-128-3p, hsa-miR-137, hsa-miR-299-5p, hsa-miR-3622a-5p, hsa-miR-486-5p, and hsa-miR-154-5p (Figure 10).

We then performed the survival analysis for each miRNA and found that longer survival time is positively correlated with hsa-miR-299-5p, hsa-miR-154, hsa-miR-299-3p, hsa-miR-31, hsa-miR-328, hsa-miR-654-5p, and hsa-miR-543 (Figure S1, *p* < 0.05). In addition, we found that the low expression of some miRNAs was associated with a higher survival rate, including hsa-miR-3622a, hsa-miR-1343, hsa-miR-24, hsa-miR-541, hsa-miR-3918, hsa-miR-224, hsa-miR-4731, hsa-miR-4726, hsa-miR-378 g, hsa-miR-4739, hsa-miR-7, hsa-miR-4640, hsa-miR-1913, hsa-miR-2467, hsa-miR-3144, and hsa-miR-5194 (Figure S2).

### 3.6. Genes Correlated with *FLAD1* in Breast Invasive Carcinoma

**Figure 11:**
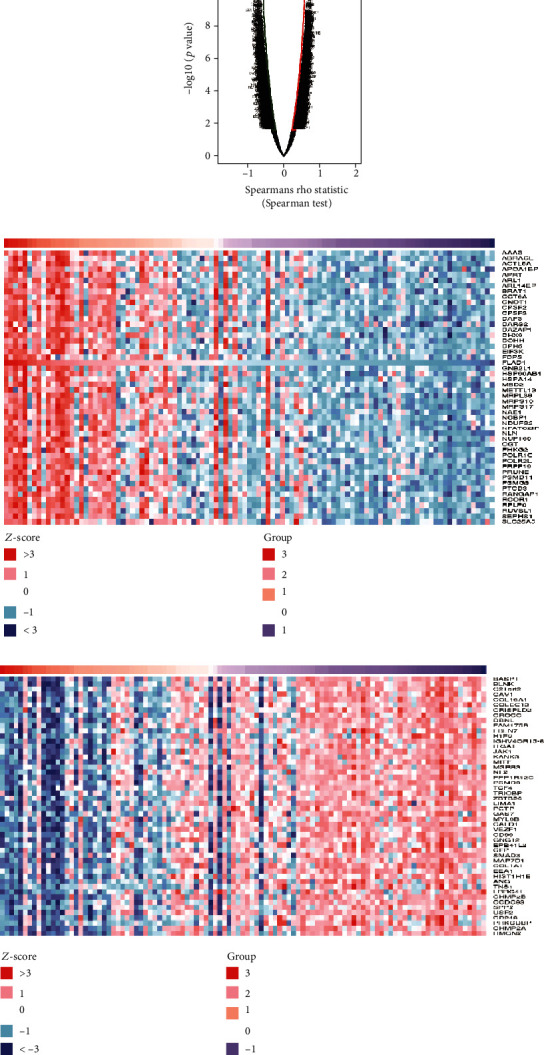
Genes differentially expressed in correlation with *FLAD1* in breast invasive carcinoma (LinkedOmics). (a) Correlations between *FLAD1* and genes differentially expressed in breast invasive carcinoma. (b, c) Heat maps showing genes positively and negatively correlated with *FLAD1*.

We used LinkedOmics to analyze proteomic data for patients with breast invasive carcinoma and found 1861 genes (dark red dots in Figure 11) showing significant positive correlations with *FLAD1* and 1870 genes (dark green dots) showing significant negative correlations, as shown in a volcano plot (Figure 11(a)) (false discovery rate [FDR] < 0.01). The top 50 genes exhibiting positive or negative correlations with *FLAD1* were evaluated in a heat map (Figures 11(b) and 11(c)).

The *FLAD1* expression showed a strong positive association with the expression of *NLN* (Spearman′s correlation = 0.69, *p* < 0.001), *UFC1* (Spearman′s correlation = 0.68, *p* < 0.001), and *UCHL5* (Spearman′s correlation = 0.67, *p* < 0.001), which function in metalloendopeptidase activity and peptide binding, UFM1 transferase activity, and endopeptidase inhibitor activity.

**Figure 12:**
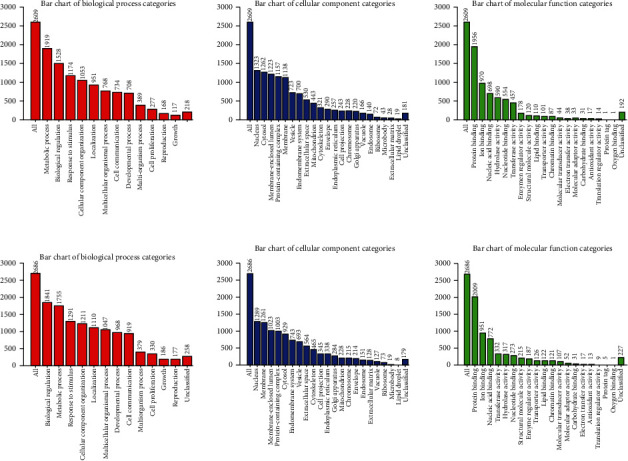
Bar charts showed KEGG pathway analysis of FLAD1 corrected genes (Linkomics). (a) KEGG pathway analysis of positive correlated genes. (B) KEGG pathway analysis of negative correlated genes.

We also conducted a KEGG analysis of positively and negatively correlated genes (Figures 12(a) and 12(b), respectively) and found the enrichment for metabolic processes, biological regulation, nucleus, and protein binding.

## 4. Discussion

*FLAD1* is related to the metabolism of water-soluble vitamins and cofactors, and *FLAD1* mutations cause a FAD synthase deficiency, which is a rare genetic disease affecting mitochondrial energy metabolism and other riboflavin metabolism [33, 34]. The overexpression of *FLAD1* has been reported in various cancers such as hepatocellular carcinoma [8], gastric cancer [10], and breast cancer [11]. In this study, we analyzed the transcription levels of *FLAD1* in pan-cancer analysis with a focus on breast cancer and further classified the results on the basis of the clinicopathologic parameters. These results provide evidences for *FLAD1* as a new biomarker of breast cancer and suggest its clinical significance.

Based on extensive database mining, *FLAD1* was found to be overexpressed in various cancers, including kidney renal papillary cell carcinoma, head-neck squamous cell carcinoma, esophageal cancers, colon adenocarcinoma, cholangiocarcinoma, breast invasive carcinoma, bladder urothelial carcinoma, and liver hepatocellular carcinoma. Amplification was the most frequent *FLAD1* alteration type identified in breast cancer. We observed higher *FLAD1* expression in the ER^−^ and PR^−^ group, HER2^+^ NPI stage 3, basal-like group, and triple-negative group. ER has two forms, *α* and *β*, which are encoded by *ESR1* and *ESR2,* respectively [35]. PR also has two different isoforms, PRA and PRB, encoded by the same *PR* gene [36]. Since ER-positive patients are eligible for hormonal therapy [37], ER status plays an important role in treatment decisions. A study based on the SEER database illustrated that patients with ER^+^PR^+^ status had better survival than those who with an ER^−^PR^−^ in each stage and age group [38]. In addition, patients with ER^+^PR^+^ breast cancer respond better to tamoxifen than ER^+^PR^−^ patients [39]. In our study, the *FLAD1* expression was correlated with ER^−^ and PR^−^, which are related to poorer survival. The basal-like group showed higher *FLAD1* expression than that in other groups. Previous studies have shown that patients with basal-like cancers have significantly worse OS and recurrence-free survival than those of their luminal A counterparts [40–42]. Similar results have been found in a breast cancer-specific survival analysis [43]. Triple-negative breast cancer lacks a therapeutic target owing to its negative profile for PR, ER, and HER2 [44]. Triple-negative breast cancer has a lower five-year survival rate compared with those of other breast cancers [45]. *FLAD1* was also related to nodal status, which is a practical parameter for estimating prognosis [46].

We further analyzed clinical parameters in breast invasive carcinoma. The expression levels were higher in patients who were 21–40 years old than at those from other age groups and were higher in the African-American group than in the Caucasian group. In addition, the *FLAD1* expression levels in the luminal subclass were significantly lower than those in the HER2-positive and triple-negative groups.

We also analyzed the significance and potential clinical application of *FLAD1*-related miRNAs. In previous studies, the *FLAD1* expression was found to be linked to let-7b with respect to tumorigenesis in breast cancer, based on the analysis of somatic single-nucleotide variants and miRNA–mRNA pairs [47]. In the present study, we found some *FLAD1*-related miRNAs, which showed significant differences in the cancer and normal samples, indicating their potential prognostic value.

We also found that the expression of *FLAD1* in breast cancer is associated with the expression of genes involved in metabolic processes, including *NLN*, *UFC1,* and *UCHL5*. Among these, *UCHL5* is reversibly recruited and activated by the 19 S proteasome and shows potential as a novel target for anticancer therapy [48]. Further research on the possibility of the application of *FLAD1* as a therapeutic target based on small-molecule probes has yielded initial results [49], indicating that FLAD1 also has certain potential as a target for cancer treatment.

This study reports the significance of *FLAD1* in cancer based on multilevel data in public databases and provides evidence for its potential as a biomarker for the diagnosis and prognosis of various cancers. One of the limitations of this study is its retrospective nature, because the analysis was only based on current databases and did not involve any prospective research for validation. In addition, this study mainly relies on bioinformatics, without a summary of detailed clinical information. Therefore, further research is needed to validate the results of this study and elucidate the biological mechanism underlying the role of *FLAD1* in cancers.

## Data Availability

The datasets in this study can be obtained from the listed database (Oncomine, cBioPortal, Breast cancer Gene-Expression Miner, UALCAN, GEO, BCIP, TNMplot, ENCORI, Kaplan-Meier Plotter and LinkedOmics).

## Supplementary Materials

Supplementary Materials Figure S1: survival analysis of positively related miRNAs. (a) hsa-miR-299-5p, (b) hsa-miR-154, (c) hsa-miR-299-3p, (d) hsa-miR-31, (e) hsa-miR-328, (f) hsa-miR-654-5p, and (g) hsa-miR-543. Figure S2: survival analysis of negatively correlated miRNAs.
