# Supplementary material for: Significant Diagnostic and Prognostic Value of FLAD1 and Related MicroRNAs in Breast Cancer after a Pan-Cancer Analysis
Source: Dis Markers. 2021 Jul 21;2021:6962526. doi: 10.1155/2021/6962526 (PMC8321750; doi:10.1155/2021/6962526)
Supplement: Supplementary Materials — Figure S1: survival analysis of positively related miRNAs. (a) hsa-miR-299-5p, (b) hsa-miR-154, (c) hsa-miR-299-3p, (d) hsa-miR-31, (e) hsa-miR-328, (f) hsa-miR-654-5p, and (g) hsa-miR-543. Figure S2: survival analysis of negatively correlated miRNAs. [file 6962526.f1.docx]

**Significant Diagnostic and Prognostic Value of FLAD1 and Related MicroRNAs in Breast Cancer after a Pan-Cancer Analysis**

Mei Mei^1,2^, Wenting Song^1^, Yingjun Wang^1^, Mingzhi Zhang^1*^

^1^ Department of Oncology, The First Affiliated Hospital of Zhengzhou University, Zhengzhou, China

^2^ The Academy of Medical Sciences, Zhengzhou University, Zhengzhou, China


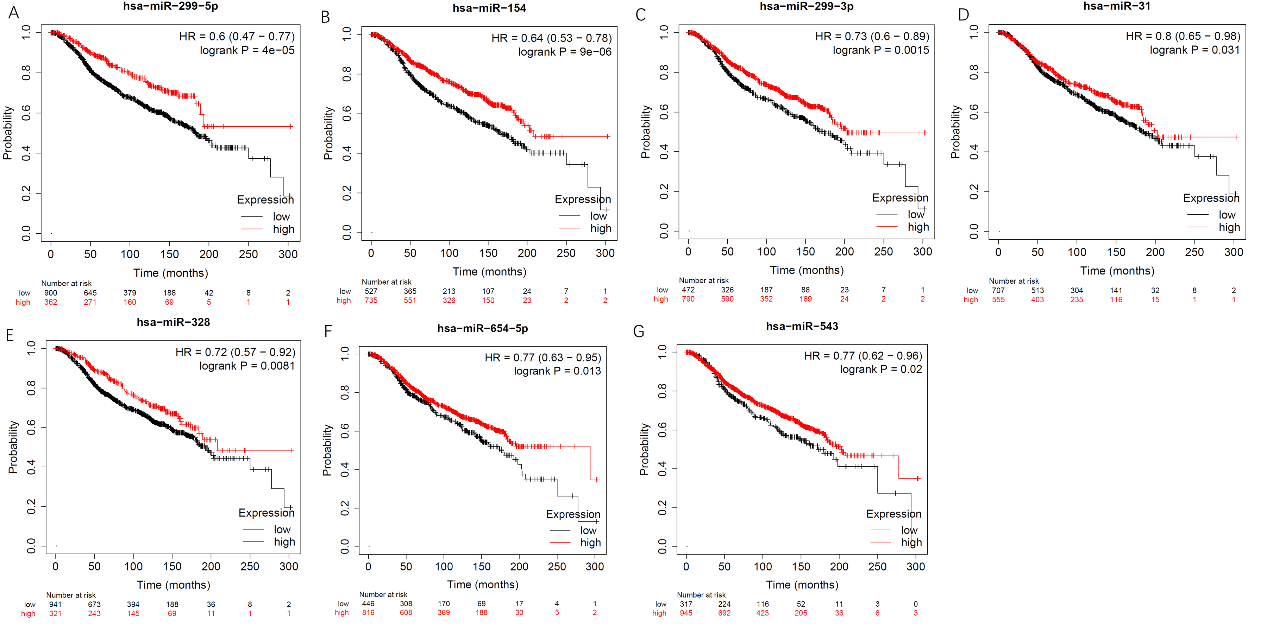


**Figure S1.** Survival analysis of positively related miRNAs. (A) hsa-miR-299-5p, (B) hsa-miR-154, (C) hsa-miR-299-3p, (D) hsa-miR-31, (E) hsa-miR-328, (F) hsa-miR-654-5p, (G) hsa-miR-543.


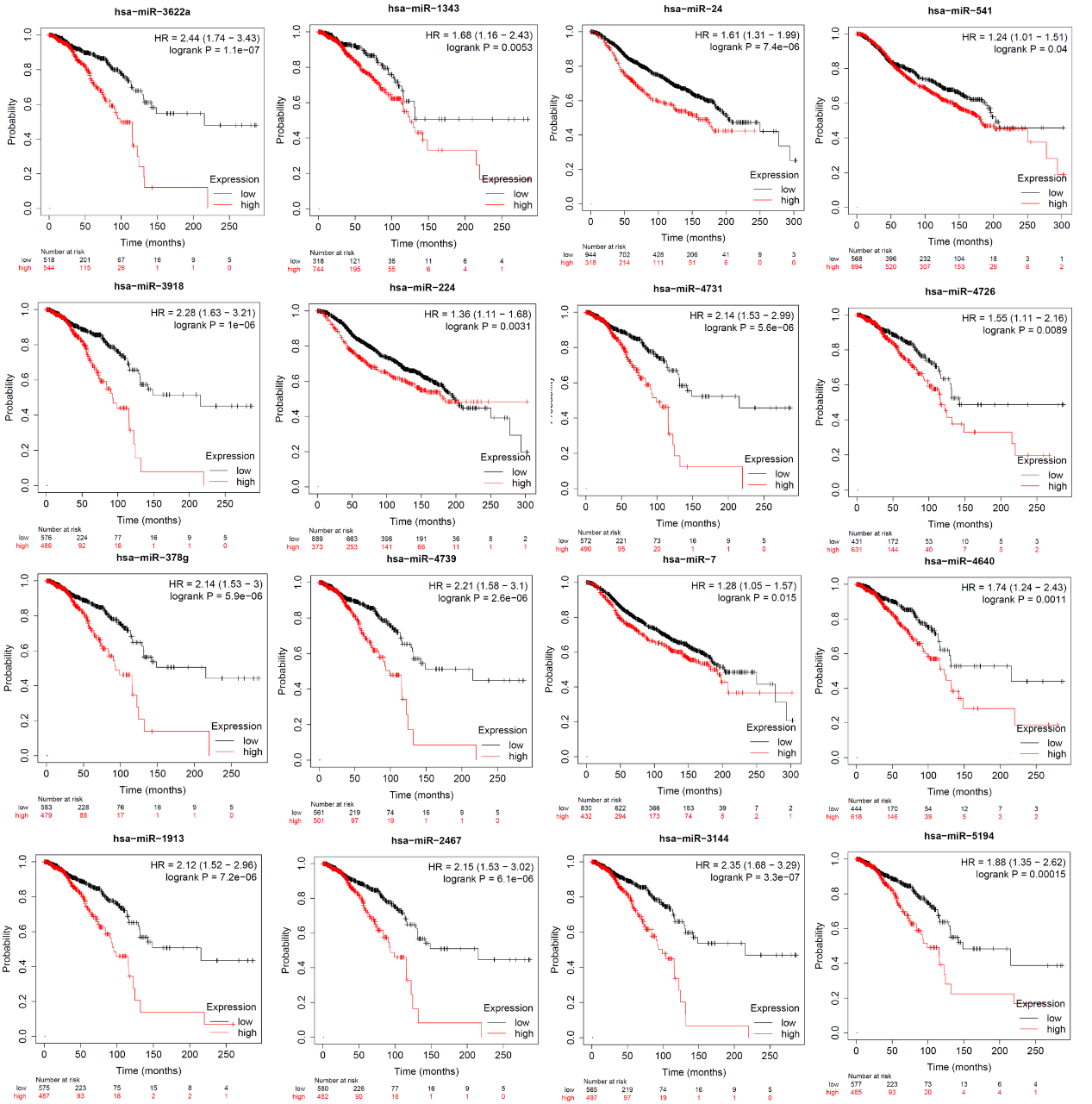


**Figure S2.** Survival analysis of negatively correlated miRNAs.
